# Lifestyle for Environment (LiFE): a global initiative to fight against climate change through community engagement and lifestyle modification

**DOI:** 10.1016/j.lansea.2023.100238

**Published:** 2023-06-13

**Authors:** Khaiwal Ravindra, Akshi Goyal, Suman Mor

**Affiliations:** a Department of Community Medicine & School of Public Health, PGIMER, Chandigarh 160012, India; b Department of Environment Studies, Panjab University, Chandigarh 160014, India

## Background

Climate change and global warming are the vital signs of anthropogenic activities severely affecting flora, fauna, ecology and human health. The intensity of climate change impacts will be determined by how fast we adopt or modify human activities for climate mitigation.

## LiFE as a tool to fight climate change

During the 26th United Nations Climate Change Conference of the Parties (COP26), India proposed "Lifestyle for Environment (LiFE) Movement" to incorporate into Nationally Determined Contributions^1^ that emphasizes the urgency of resolving the crisis faced by our planet through human-centered, communal efforts and strenuous action that promotes sustainable development.

Instead of "mindless and wasteful consumption", the concept advocates for an environment-friendly conscious lifestyle that emphasizes "mindful and purposeful utilization". By 2028, at least 80% of India's villages and urban local bodies are expected to be environmentally friendly. Mission LiFE democratizes the battle against climate change by motivating everyone to do every possible thing in their daily lives to safeguard the environment by modifying their lifestyles and advancing global health.

## LiFE and global engagement

LiFE targets to promote international partnerships on a global scale. This initiative extends beyond specific regions, encompassing countries from different continents. There is a worldwide call to bring flagship solutions or strategies to provide the groundwork for behavior change toward more sustainable lifestyles in the next few decades. Mission LiFE collectively proposes three-tier fundamental shifts to attain sustainability: Phase-I (changing demand), Phase-II (changing supply) and Phase-III (changing policy).

**Fig. 1:**
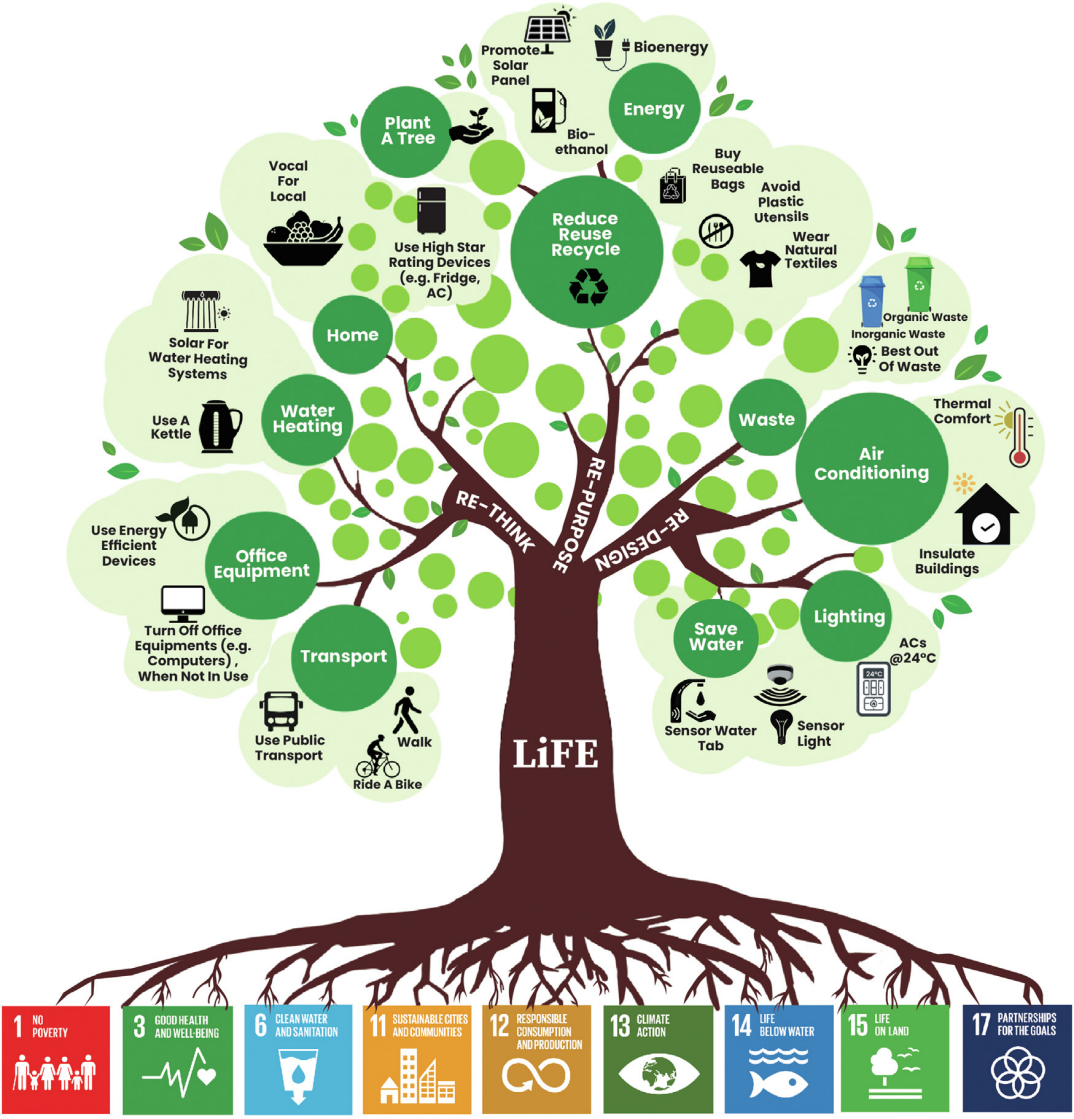
LiFE: Citizen-oriented approaches to mitigate climatic impacts.

LiFE aims to form and foster a worldwide community of eco-conscious people known as "Pro-Planet People", i.e., P3 community where an ecosystem promotes and enables environmentally responsible behavior to be self-sustaining^1^ via the domino effect (Fig. 1). Numerous studies indicate that practicing an action for at least 21–66 days is necessary to become a habit.^2^^,^^3^ Therefore, the LiFE 21-Day Challenge is introduced to enable citizens to do one simple eco-friendly activity/day for 21 days and subsequently adopt an eco-friendly lifestyle.

## LiFE and its integration with other initiatives

LiFE is not only to adopt a zero-carbon lifestyle but to make sustainable choices in day-to-day life, with the utmost focus on the fundamental principles of the 3R's, i.e., re-think, re-purpose, re-design. Mission LiFE will sync directly and indirectly with every Sustainable Development Goal (SDG), mainly SDG1, SDG3, SDG6, SDG11, SDG12, SDG13, SDG14, SDG15 and SDG17.

By nudging and encouraging human behavior change, mission LiFE will also address the various other climate-friendly approaches taken by India, National Action Plan on Climate Change (NAPCC), National Green Hydrogen Mission, National Clean Air Programme (NCAP), Green Credit Programme, PM-Panchamrit, PRANAM programme, Namami Gange Programme (Clean Ganga), Pradhan Mantri Ujjwala Yojana (Clean household fuels), GOBARdhan Scheme (Cattle waste or other biodegradable waste into compressed biogas), Nagar Van Scheme (Urban Forest Scheme), Jal Jeevan Mission (Safe Drinking Water Mission), Atal Bhujal Yojana (ABHY-Groundwater Management Scheme), Green Skill Development Programme (GSDP), Swachh Bharat Mission (Clean India Mission), and Catch the Rain campaign.

## LiFE and future challenges

Carbon emissions are dominated by industries, but individual environmentally conscious choices and national/international policies can restrict practices that emit carbon, e.g., fossil fuel phase-out. Hence, the battle against climate-related disasters also requires broader support from people and communities at the grassroots level, as they play a significant role in fostering sustainable behaviors and promoting environmental stewardship at the local level.

The next COP28 in UAE offers a crucial political chance to establish trust and quicken progress on all aspects of the Paris Agreement. India can play an essential bridging role because of its vast economy and climatic susceptibility. Additionally, India's G20 presidency has the chance to usher into a new age of sustainability consistent with its history, culture, and tradition.

## Contributors

Khaiwal Ravindra: Conceptualization, Writing - review & editing, Visualization.

Akshi Goyal: Writing - review & editing.

Suman Mor: Supervision, Formal analysis, Writing - review & editing.

## Declaration of interests

This work is linked with a CEEP project funded by MoEF&CC, India. However, this work is developed and planned independently. The authors declare no conflict of interest.
